# A Lightweight Attention-Based CNN Model for Efficient Gait Recognition with Wearable IMU Sensors

**DOI:** 10.3390/s21082866

**Published:** 2021-04-19

**Authors:** Haohua Huang, Pan Zhou, Ye Li, Fangmin Sun

**Affiliations:** 1 Joint Engineering Research Center for Health Big Data Intelligent Analysis Technology, Shenzhen Institute of Advanced Technology, Chinese Academy of Sciences, Shenzhen 518055, China; hh.huang@siat.ac.cn (H.H.); pan.zhou@siat.ac.cn (P.Z.); ye.li@siat.ac.cn (Y.L.); 2 University of Chinese Academy of Sciences, Beijing 100049, China

**Keywords:** gait recognition, lightweight model, wearable devices, attention mechanism, CNN

## Abstract

Wearable sensors-based gait recognition is an effective method to recognize people’s identity by recognizing the unique way they walk. Recently, the adoption of deep learning networks for gait recognition has achieved significant performance improvement and become a new promising trend. However, most of the existing studies mainly focused on improving the gait recognition accuracy while ignored model complexity, which make them unsuitable for wearable devices. In this study, we proposed a lightweight attention-based Convolutional Neural Networks (CNN) model for wearable gait recognition. Specifically, a four-layer lightweight CNN was first employed to extract gait features. Then, a novel attention module based on contextual encoding information and depthwise separable convolution was designed and integrated into the lightweight CNN to enhance the extracted gait features and simplify the complexity of the model. Finally, the Softmax classifier was used for classification to realize gait recognition. We conducted comprehensive experiments to evaluate the performance of the proposed model on whuGait and OU-ISIR datasets. The effect of the proposed attention mechanisms, different data segmentation methods, and different attention mechanisms on gait recognition performance were studied and analyzed. The comparison results with the existing similar researches in terms of recognition accuracy and number of model parameters shown that our proposed model not only achieved a higher recognition performance but also reduced the model complexity by 86.5% on average.

## 1. Introduction

Recent years witnessed a remarkable growth in the variety and number of Wearable Intelligent Devices (WID). The application of WID becomes more and more pervasive, including mobile payment, instant messaging, social networking and entertainment, positioning and navigation, telecommuting and health monitoring etc. Along with the great convenience brought by the WID, comes the high privacy leakage risk. Due to the large amount of private information stored in or collected by the WID, the security of the WID is of great importance.

Nowadays, biometrics have become the most popular technology for access control of the WID [1]. Biometrics recognize individual identity through the distinctive, stable and measurable physiological or behavioral characteristics of human [2]. There are mainly two kinds of biometrics, namely physiological biometrics and behavioral biometrics. Physiological biometrics are related to the shape of the body, e.g. human face [3], fingerprint [4] and iris [5], etc.; while behavioral biometrics are related to the pattern of behavior of a person, including gait, keystroke, signature, etc.

Although physiological biometrics have been widely used, they have many insurmountable shortcomings. Firstly, the sensors for physiological features acquisition (e.g., fingerprint scanner, camera) are expensive and large in size, which make it impossible to integrated them into the low-cost and small size WID. Secondly, physiological biometrics, like fingerprint, is easy to be copied and tampered [6]. Lastly, due to the static nature of physiological characteristics, physiological biometrics often require the user’s explicit interaction with the device, and cannot achieve active, real-time and continuous user identification [7], which would be a risk if the unlocked device gets lost [8].

Gait is a kind of behavior biometrics, which refers to the walking posture of a subject [9]. Previous studies have shown that the uniqueness of each person’s gait can hardly be imitated or copied [1,2,3]. Gait based identity recognition (gait recognition) is an active, real-time and continuous identity recognition method [7,8], which doesn’t need the explicit interaction of the users and has high security. Besides, with the development of the microelectronics technology, almost all WID have integrated with Inertial Measurement Unit (IMU) for their low cost, small size and low power consumption [10]. This makes it possible to acquire the gait information using the IMU that built-in WID to authenticate or recognize the users of the WID.

In this paper, we proposed a lightweight attention-based Convolutional Neural Networks (CNN) model for efficient gait recognition with the wearable IMU sensors. The outstanding ability of the CNN for biometric recognition have been proven in previous studies [1,3,5]. We improved the model by reducing the model complexity to make it suitable for WID and improving the gait recognition robustness to make it suitable for real-word scenarios. The main innovations and contributions of this study are summarized as follows:

(1) We proposed a new attention method that can reduce model parameters based on contextual encoding information [11] and depthwise separable convolution [12]. The lightweight CNN with the proposed attention mechanism can extract more distinctive gait features and improve recognition accuracy.
(2) We conducted comprehensive experiments on two public datasets: OU-ISIR dataset, which is the largest public gait dataset containing the maximum number (744) of participants, and whuGait dataset, the gait data which were collected in real-world unconstrained scenarios. The impact of different data segmentation methods and attention mechanisms on gait recognition performance were studied and analyzed.

The following structure of this paper is scheduled as follows: The related works on gait recognition are described and discussed in Section 2; the proposed lightweight attention-based CNN model for gait recognition is introduced in Section 3; then, the experiments on public gait datasets and corresponding performances are described in Section 4; and finally, conclusions and future works are given in Section 5.

## 2. Related Works

Existing studies on IMU-based gait recognition can be mainly categorized into two kinds: template matching methods and machine learning methods [13,14]. For template matching methods, the user’s identity is recognized by calculating and comparing the similarity between the gait templates stored in the WID with the gait cycle need to be detected [15]. If the similarity is higher than the predefined threshold the user is recognized as the legitimate user. Methods used for calculating similarity mainly include Dynamic Time Warping (DTW) [16], Pearson Correlation Coefficient (PCC) [17] and Cross-Correlation [18], etc. Various kinds of template matching methods have been studied by many previous works [19,20,21,22,23,24,25], and good performance has been achieved under laboratory conditions. However, the above mentioned gait template matching methods need to detect the gait cycles to construct the gait template and test samples. Gait cycle detection is a challenging work since it is sensitive to noise and device positions [26,27,28] and changes in pace, road conditions and device position are likely to cause gait cycle detection failures or inter-cycle phase misalignment [23,26], which will lead to wrong recognition decisions.

Machine learning methods realized gait recognition by extracting and classifying the unique features of gait signals into different classes [29,30,31]. Previous studies used support vector machines (SVM) [32,33,34], nearest neighbors (KNN) [35,36,37] and random forests (RF) [32,33] for gait identification, and achieved more robust performance than template matching methods. However, the recognition accuracy of these models were greatly affected by the manually extracted features. At present, there is no standard on how to manually extract distinctive gait features [38]. Extracting features manually requires researchers to have rich professional knowledge and experience in related fields, and must go through data preprocessing, feature engineering and continuous experimental verification and improvement to obtain good results, which make it time-consuming and difficult [39].

Recent studies [13,14,27,38,40,41,42] indicate that the use of deep learning techniques to study gait recognition has become a new promising trend. Deep learning networks have strong nonlinear representation learning capabilities, and it can automatically extract data features for classification and other tasks [43]. The authors [13] was the first who used the Convolutional Neural Networks (CNN) for gait recognition in 2016. They designed a IDNet framework based on CNN and One-class SVM [44] for user identification and authentication using the accelerometers and gyroscopes data collected by smart phones. Gait features were first extracted through a three-layer CNN, then the PCA [45] algorithm was used to reduce the dimension of the features. Later, the features were input into the One-class SVM for user identification and authentication. Their results showed that CNN has learned more useful features automatically and achieved better performance when compared to manual feature extraction. Since then, a large number of deep learning-based gait recognition methods have been proposed in [14,27,38,40,41,42]. These methods have made extensive comparisons with traditional machine learning algorithms and template matching algorithms, and have achieved better and robust improvement in recognition accuracy. But there is an obvious problem with them, that is, the models have high complexity (large amount of model parameters), which is not suitable for wearable smart devices with limited computing power and capacity.

In order to solve the problem of high model complexity of the existing gait recognition network based on deep learning, we proposed a lightweight attention-based CNN model for gait-based identification using wearable IMU sensors in this research work. Specifically, a four-layer lightweight CNN was first employed to extract gait features. Then, an attention mechanism based on contextual encoding information [11] and depthwise separable convolution [12] was proposed to strengthen the gait features and reduce model parameters, the number of model parameters determines the model complexity and the size of the memory occupied by the model. Finally, the Softmax classifier was used for classification based on the enhanced features and output decision results. The experiments were conducted on two public datasets whuGait and OU-ISIR, and the recognition accuracy were improved by our lighter model on real scenarios and the largest population datasets compared with existing schemes [27,42].

## 3. Methods and Materials

### 3.1. Datasets

In this paper, two public gait datasets: OU-ISIR dataset and whuGait dataset were used. The OU-ISIR dataset is the largest public gait dataset containing the maximum number (744) of participants provided by Ngo et.al from Osaka University [46]. While, whuGait dataset provided by Zou et.al from Whuhan University was collected by the inertial sensors in smartphones in the wild under unconstrained conditions without knowing when, where, and how the user walks. Both of the datasets have been preprocessed and shared by Zou et.al in their studies [42] which was published in 2020. Detail information of the datasets used in our study was proposed as follows.

whuGait dataset: The whuGait dataset contains inertial data of 118 subjects collected by smart phones in a totally unconstrained condition without knowing when, where and how the participants walk. The sampling rate of the sensors is 50 Hz and each sample contains 3-axis accelerometer data and 3-axis gyroscope data. The whuGait dataset consists of 8 sub-datasets, the Datasets #1 to #4 are for person identification, the Datasets #5 and #6 are for authentication, the Datasets #7 and #8 are used for separating walking data from non-walking data [42]. So only Datasets # 1 to #4 are used in this paper for gait identification. The main differences among the four datasets include the number of participants, data segmentation method, whether the samples overlap or not, samples size, etc. The detail information about Dataset #1 to #4 are listed in Table 1.

OU-ISIR dataset: The OU-ISIR dataset has been the largest inertial sensor-based gait database with the maximum number of participants. This dataset includes 744 subjects (389 males and 355 females) with wide range of ages from 2 to 78 years, making it a realistic scenario. The Gait signals are captured at 100 Hz from center IMU mounted on a waist belt. Each of the 744 persons walked for 9 meters on a flat surface, and there are few inertial gait data for any single subject (18.73 seconds at most and 5.61 seconds at least with a sensor frequency of 100 Hz) [42]. The details are listed in Table 1.

**Figure 1:**
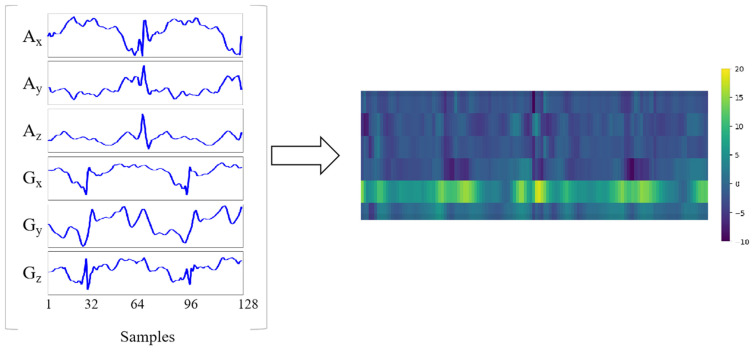
One input sample. A_x_, A_y_, A_z_ are the 3-axis acceleration data, and G_x_, G_y_, G_z_ are the 3-axis angular velocity data. The 6-axis sensor data are combined into a matrix with the shape of 6 × 128.

For the two datasets used in this paper, the 3-axis acceleration data and the 3-axis angular velocity data are combined into a matrix with the shape of 6x 128 as the input of the model. By treating the matrix as an image with one channel, the network can extract the gait features for classification. The visualization of one input sample is shown in Figure 1.

### 3.2. Methods

**Figure 2:**
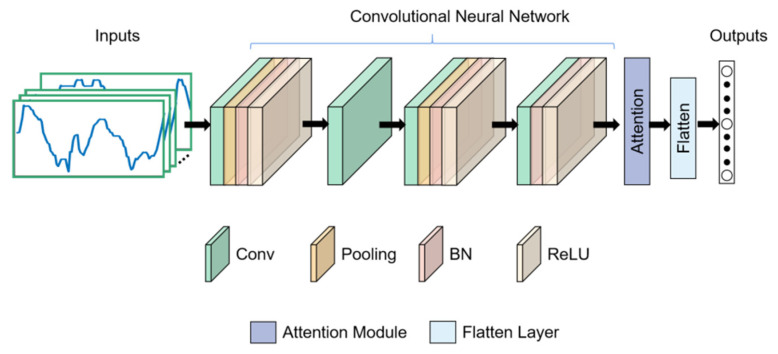
The architecture of gait identification network.

The architecture of our proposed gait recognition method is shown in Figure 2. The model includes a four-layer lightweight CNN and an attention module. The CNN was used to extract gait features at the forefront of the entire network. The attention module based on contextual encoding layer [11] and depthwise separable convolution [12] are used to enhance the gait features related to the identity category and reduce the model parameters. Finally, the enhanced features are flattened into a one-dimensional vector and fed to the Softmax classifier for identify recognition.

#### 3.2.1. Lightweight CNN

The network structure and the parameters of the proposed lightweight CNN is shown in Table 2. As the main function of the proposed lightweight CNN is to automatically extract gait features and feature optimization task is left to the attention module, which is main focus in our method, we adopt the four layer CNN proposed by [42] and improved it by adding a BatchNormalization (BN) layer [47] and a ReLU layer [48] to accelerate the speed of network training and convergence, and prevent gradient disappearance and result overfitting.

#### 3.2.2. Attention Module

The outstanding performance of the attention mechanism in the application of image recognition, speech recognition, ECG signal processing and natural language processing, etc. has been proven by [49,50]. In this paper, we used the channel attention mechanism proposed by [51] to learn the correlations between channels, according to the learned correlations, the weights of different channels were computed and multiplied with the original feature maps to enhance the feature maps of important channels [52]. The correlations are usually realized by using a global average pooling layer [51] and a fully connected layer, however, this greatly increased the parameters of the network. To make the model lightweight, we proposed a new method to calculate the correlations between channels without increasing the network parameters.

Let the output of the lightweight CNN be $\in R^{H\times W\times C}$, where *H*, *W* and *C* are height, width and channel dimension, and the weight of *i*-th channel is represented as Equation (1).

(1) $$w_{i}=\frac{F_{i}}{\sum_{j=1}^{C}F_{j}}$$

where i=1,2,…,C. $F_{i}$ represents the contextual encoding information of the *i*-th channel. $\overset{C}{\underset{j=1}{\sum }}F_{j}$ represents the sum of the contextual encoding information of all channels.

In our method, we used the context encoding layer (CEL) proposed by literature [11] to obtain the contextual encoding information. The CEL contains K encoding vectors that are D-dimensional, and the values of these encoding vectors are initialized as random decimals and gradually optimized during the network training process. The CEL will output K coded values for each channel and the number of the output coded values only related to K. We can use the result of the sum of K coded values or the coded value obtained by setting K = 1 as the contextual encoding information of each corresponding channel. In our method, we set K = 1.

In addition to the CEL method proposed for computing the weights of channels, we also used depthwise separable convolution [12] to further remove the redundant features in the feature maps of the important channels. The channel attention mechanism can detect which channels are important and enhance their feature maps. However, the channel attention mechanism ignored that there may still be some redundant features in the feature maps of the important channels. To eliminate the redundant features, we further used the depthwise separable convolution to extract features from the feature map of each channel before multiplying the original feature maps with the weights of channels.

Depthwise separable convolution, proposed by [12], can make the CNNs more lightweight, it performs convolution operations within a single channel so that it doesn’t change the number of channels, i.e., doesn’t need a specific number of filters, which reduces the number of network parameters.

**Figure 3:**
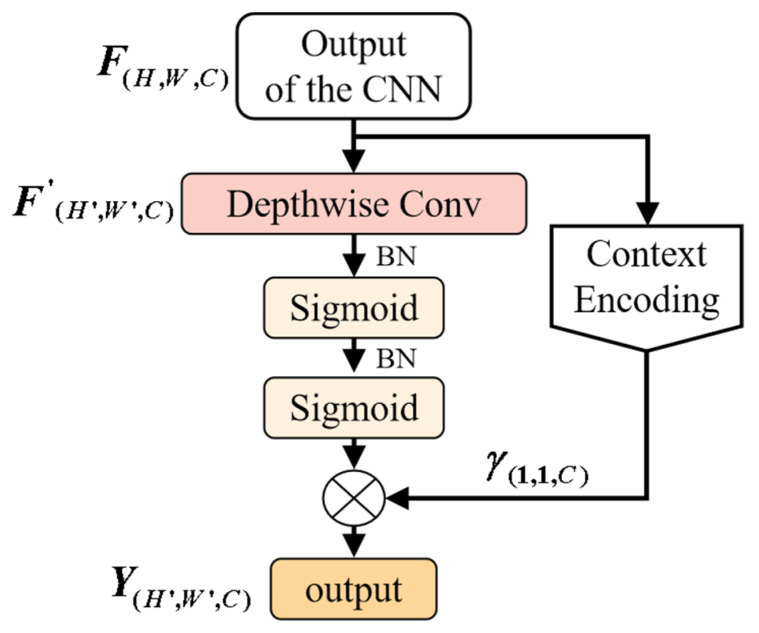
Structure of proposed attention mechanism.

We further proposed a new channel attention method named CEDS based on the Equation (1), CEL and depthwise separable convolution module, which can be denoted as the Equation (2).

(2) $$Y_{\left(H^{\prime },W^{\prime },C\right)}=\delta_{N}\left(\delta_{N}\left(D_{c}\left(F_{\left(H,W,C\right)}\right)\right)\right)⊗\gamma_{\left(1,1,C\right)}$$

where $D_{c}\left(\cdot \right)$ represents depthwise separable convolution operation, in this paper, the kernel size of depthwise separable convolution is set to 1x3; $\delta_{N}\left(\cdot \right)$ is the BN+Sigmoid. $\gamma_{\left(1,1,C\right)}$ represents the weights of channels calculated by Equation (1) and CEL. $F_{\left(H,W,C\right)}$ is the output of the lightweight CNN. The structure of the proposed CEDS is shown in Figure 3.

## 4. Experimental Results

### 4.1. Experimental Methods and Evaluation Metrics

In the model training process, the learning rate of the network was set to 0.0001 and the batch size was set to 512. Additionally, 20% of the training data is divided into the validation set, and the early stopping method [53] was used to control the times of iteration during training. The early stopping method can be used to end the network training when the performance, like accuracy and loss, of the network on the validation set has no improvement for N consecutive times. It can save the model with the best performance on the validation set during the training process. The best model can solve the network overfitting problem and improve the generalization performance, when using it to predict the test set.

In this paper we set N = 30. For comprehensive evaluations, we used four metrics, namely Accuracy, Recall, F1-score and AUC (Area under the ROC Curve). Higher scores of these metrics indicated better performance.

### 4.2. Experimental Results

In this part, we conducted multiple comparative experiments to prove the effectiveness of our method.

#### 4.2.1. Performance Comparison of Model with and without Attention Mechanism

Our method proposed in this paper is a network based on the attention mechanism. In order to verify the effectiveness of the proposed attention method, we compared the performance of the gait recognition model with or without attention mechanism, and the experimental results are shown in Table 3.

Firstly, it is obvious that the CNN with CEDS attention has achieved a improvement in Accuracy, Recall and F1-score with fewer network parameters, especially in OU-ISIR dataset, the average improvement of the three evaluation metrics is about 39.5%. Secondly, our method has a more significant performance improvement on these datasets (Dataset #3, Dataset #4 and OU-ISIR) using fixed length based gait segmentation method compared to the datasets (Dataset #1, Dataset #2) using gait cycle-based data segmentation method. As gait cycle detecting methods are noise-sensitive and position-dependent, it is still a challenging work to accurately detect the gait cycles. Fixed length based data segmentation method doesn’t need to detect gait cycles, which makes it a simple and efficient way for gait signal segmentation in practical applications.

#### 4.2.2. Performance Comparison of Different Attention Mechanisms

In this part, we compared the performance of the CEDS attention proposed in this paper with the well-known Squeeze-and-Excitation (SE) attention proposed in [51] to further testify the effectiveness of the proposed algorithm. The test results are shown in Table 4, we can see that the recognition performance of the CEDS attention proposed in this paper is superior to the SE attention.

#### 4.2.3. Performance Comparison with Existing Research Results

Our method was then compared with the existing similar works proposed in [42] and [27]. Tran, et.al [27] claimed that they have improved the gait recognition performance compared with literature [42]. Their method compared the template matching methods with traditional machine learning methods, and the results obtained got the best gait recognition performance on whuGAIT and OU-ISIR datasets by till now.

The results of the comparative experiments are shown in Table 5. Taking the performance of [42] as the baseline, we can see that our proposed method has a higher performance improvement than [27] on both Dataset #1 and OU-ISIR. Besides, our proposed model not only achieved the accuracy improvement, but also reduced the number of network parameters by 86.5% on average compared with that proposed by [42]. A lightweight model with higher recognition accuracy is important for wearable devices in real scene.

According to the above comparative analysis, our method outperformed the existing methods and has superior gait recognition performances.

#### 4.2.4. Discriminative Features Comparison of Different Methods

The method proposed in this paper improved the recognition performance on both whuGait and OU-ISIR datasets with a more lightweight model. This reflected that the features enhanced by CEDS attention method are more discriminative. In order to prove this point of view, we visualized the extracted gait features outputted by the last layer of the network proposed use the t-SNE [54] algorithm.

**Figure 4:**
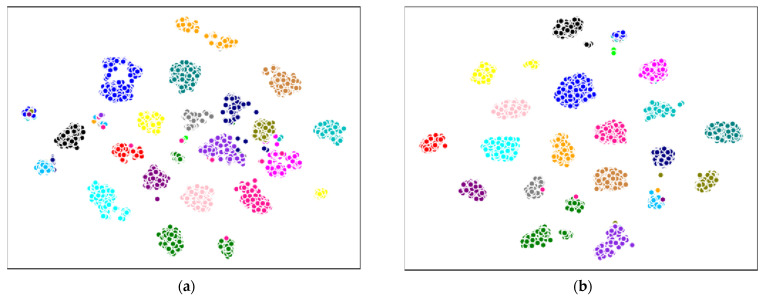
Gait features visualization of the 20 subjects in Dataset #2 (**a**) features extracted by CNN and (**b**) features extracted by our proposed CNN+CEDS. The dots of different colors represent the extracted gait features of different subjects.

As the number of subjects in the other datasets are too large to do the visualization, we did the dimensionality reduction visualization experiment only on Dataset #2 of whuGait dataset which included 20 subjects. The experimental results are shown in Figure 4. The dots of different colors represent the extracted gait features from different subjects, one can see from the results that our proposed CNN+CEDS method decreased the intra-class distance as well as increased the inter-class distance compared with the CNN method. The gait feature visualization results testified the advantage of the proposed CNN+CEDS gait feature extraction method.

## 5. Conclusions and Future Work

In this paper, a lightweight CNN model with attention mechanism was proposed for gait recognition using wearable sensors. Two public datasets containing gait signals collected in the wild and gait signals of the largest population were used to testify the robustness, accuracy and lightweight of the proposed methods. The proposed CEDS attention module enhanced the distinctness of the extracted gait features and reduced the network parameters.

The performance improvement of our method was obvious on OU-ISIR dataset. According to the four comparative experiments, the smallest performance improvement was 7.37% on accuracy shown in Table 5, and the largest performance improvement was 42.8% on F1-score shown in Table 3.

The proposed method also achieved a better performance compared with [42] and [27] on whuGAIT dataset. Although the performance improvement seems relatively small on the Dataset #1 and Dataset #2 that were collected in the wild, the number of network parameters of our proposed method was 86.5% less than that of [42], which was important for wearable devices in practical applications. Besides, it was worth to note that, our method has a more significant performance improvement on dataset #3 and dataset #4, which used fixed length based data segmentation method. Fixed length based data segmentation method avoids detecting gait cycles, which makes it simple and efficient in practical applications of wearable devices.

For the future work, we would further implement our gait recognition model on a smartphone and testify the gait recognition performance including response time, usage of mobile computing and storage resources, energy consumption, etc. in various real scenarios. Besides, using the gait information collected from different human positions, such as the wrist, gait recognition research is also a work point. We also consider gait recognition in case of injury or physical exhaustion.

## Figures and Tables

**Table 1:** Information of the datasets used in this paper [42].

Dataset Name	Number of Subjects	Data Segmentation Method	Overlap of Samples	Samples for Training	Samples for Test
Dataset #1	118	Gait cycle based segmentation (two gait cycles as a sample)	50%	33,104	3740
Dataset #2	20	Gait cycle based segmentation (two gait cycles as a sample)	0	44,339	4936
Dataset #3	118	Fixed length based segmentation (sample length = 128)	50%	26,283	2991
Dataset #4	20	Fixed length based segmentation (sample length = 128)	0	35,373	3941
OU-ISR	744	Fixed length based segmentation (sample length = 128)	61%	13,212	1409

Note: (1) For gait cycle based segmentation, a single sample is interpolated into a fixed length of 128 (using Linear Interpolation function). (2) There is no overlap between the training set and test set. (3) The datasets used in this paper is available.at https://github.com/qinnzou/ (accessed on 15 December 2020).

**Table 2:** Structure and parameters of the lightweight CNN.

Layer Name	Kernel Size	Kernel Num.	Feature Map
Conv1	1 × 9	32	6 × 64 × 32
Pool1	1 × 2	/	6 × 32 × 32
BN	/	/	6 × 32 × 32
ReLU	/	/	6 × 32 × 32
Conv2	1 × 3	64	6 × 32 × 64
Conv3	1 × 3	128	6 × 32 × 128
Pool2	1 × 2	/	6 × 16 × 128
BN	/	/	6 × 16 × 128
ReLU	/	/	6 × 16 × 128
Conv4	6 × 1	128	1 × 16 × 128
BN	/	/	1 × 16 × 128
ReLU	/	/	1 × 16 × 128

**Table 3:** Comparison of the network with and without attention mechanism.

Dataset Name	Classification Methods	Accuracy	Recall	F1-Score	Parameters Num.
Dataset #1	CNN	93.96%	93.95%	93.21%	372,598
	CNN+CEDS (Ours)	94.71%	94.67%	93.98%	344,055
Dataset #2	CNN	97.21%	94.96%	94.89%	171,796
	CNN+CEDS (Ours)	97.67%	95.51%	95.37%	168,341
Dataset #3	CNN	92.88%	92.02%	90.90%	372,598
	CNN+CEDS (Ours)	95.09%	95.26%	94.45%	343,543
Dataset #4	CNN	97.97%	96.50%	96.87%	171,796
	CNN+CEDS (Ours)	98.58%	97.38%	97.81%	168,341
OU-ISIR	CNN	59.62%	58.69%	53.40%	1,657,321
	CNN+CEDS (Ours)	97.16%	96.96%	96.20%	1,468,266

**Table 4:** Comparison of different attention mechanisms.

Dataset Name	Methods	Accuracy	Recall	F1-Score
Dataset #1	CNN+SE	94.20%	93.99%	93.13%
	CNN+CEDS (Ours)	94.71%	94.67%	93.98%
Dataset #2	CNN+SE	97.24%	94.93%	94.76%
	CNN+CEDS (Ours)	97.67%	95.51%	95.37%
Dataset #3	CNN+SE	93.38%	93.16%	92.10%
	CNN+CEDS (Ours)	95.09%	95.26%	94.45%
Dataset #4	CNN+SE	98.05%	96.33%	96.78%
	CNN+CEDS (Ours)	98.58%	97.38%	97.81%
OU-ISIR	CNN+SE	60.04%	58.65%	54.20%
	CNN+CEDS (Ours)	97.16%	96.96%	96.20%

**Table 5:** Comparative Analysis.

Dataset Name	Classification Methods	Accuracy	AUC	Parameters Num.	Memory Size Needed
Dataset #1	CNN+LSTM [42]	93.52%	-	4,716,406	56.7 Mb
	LSTM & CNN [27]	94.15%	-	-	-
	CNN+CEDS (Ours)	94.71%	94.81%	344,055	4.24 Mb
Dataset #2	CNN+LSTM [42]	97.33%	-	4,415,252	53.1 Mb
	CNN+CEDS (Ours)	97.67%	97.96%	168,341	2.13 Mb
OU-ISIR	LSTM [42]	72.32%	-	4,986,601	59.9 Mb
	LSTM & CNN [27]	89.79%	-	-	-
	CNN+CEDS (Ours)	97.16%	97.32%	1,468,266	17.7 Mb

Note: Since Tran, et al. [27] did not share their source codes, we did not compare the number of model parameters with them.

## References

[1] Peinado-Contreras A., Munoz-Organero M. (2020). Gait-Based Identification Using Deep Recurrent Neural Networks and Acceleration Patterns. Sensors.

[2] Shen W., Tan T. (1999). Automated biometrics-based personal identification. Proc. Natl. Acad. Sci. USA.

[3] He R., Wu X., Sun Z., Tan T. (2018). Wasserstein CNN: Learning Invariant Features for NIR-VIS Face Recognition. IEEE Trans. Pattern Anal. Mach. Intell..

[4] Zheng G., Yang W., Valli C., Qiao L., Shankaran R., Orgun M.A., Mukhopadhyay S.C. (2018). Finger-to-heart (F2H): Authentication for wireless implantable medical devices. IEEE J. Biomed. Health Inform..

[5] Wang K., Kumar A. (2019). Toward more accurate iris recognition using dilated residual features. IEEE Trans. Inf. Forensics Secur..

[6] Zhang H., Liu J., Li K., Tan H., Wang G. (2020). Gait learning based authentication for intelligent things. IEEE Trans. Veh. Technol..

[7] Zhang S., Liu J. (2018). Analysis and optimization of multiple unmanned aerial vehicle-assisted communications in post-disaster areas. IEEE Trans. Veh. Technol..

[8] Derawi M.O., Nickel C., Bours P., Busch C. Unobtrusive User-Authentication on Mobile Phones Using Biometric Gait Recognition. Proceedings of the Sixth International Conference on Intelligent Information Hiding and Multimedia Signal Processing (IIH-MSP).

[9] Stevenage S.V., Nixon M.S., Vince K. (1999). Visual Analysis of Gait as a Cue to Identity. Appl. Cogn. Psychol..

[10] Zang W., Zhang S., Li Y. (2016). An accelerometer-assisted transmission power control solution for energy-efficient communications in WBAN. IEEE J. Sel. Areas Commun..

[11] Zhang H., Xue J., Dana K. Deep TEN: Texture Encoding Network. Proceedings of the IEEE Conference on Computer Vision and Pattern Recognition (CVPR).

[12] Chollet F. Xception: Deep Learning with Depthwise Separable Convolutions. Proceedings of the IEEE Conference on Computer Vision and Pattern Recognition (CVPR).

[13] Gadaleta M., Rossi M. (2018). Idnet: Smartphone-based gait recognition with convolutional neural networks. Pattern Recognit..

[14] Qin Z., Huang G., Xiong H., Choo K.-K.R. (2019). A fuzzy authentication system based on neural network learning and extreme value statistics. IEEE Trans. Fuzzy Syst..

[15] Deb S., Yang Y.O., Chua M.C.H., Tian J. (2020). Gait identification using a new time-warped similarity metric based on smartphone inertial signals. J. Ambient. Intell. Humaniz. Comput..

[16] Müller M. (2007). Dynamic time warping. Inf. Retr. Music Motion.

[17] Adler J., Parmryd I. (2010). Quantifying colocalization by correlation: The Pearson correlation coefficient is superior to the Mander’s overlap coefficient. Cytom. Part A.

[18] Wren T.A.L., Do K.P., Rethlefsen S.A., Healy B. (2006). Cross-correlation as a method for comparing dynamic electromyography signals during gait. J. Biomech..

[19] Sun H., Yuao T. (2012). Curve aligning approach for gait authentication based on a wearable accelerometer. Physiol. Meas..

[20] Sun F., Mao C., Fan X., Li Y. (2018). Accelerometer-based speed-adaptive gait authentication method for wearable IoT devices. IEEE Internet Things J..

[21] Sun F., Zang W., Gravina R., Fortino G., Li Y. (2020). Gait-based identification for elderly users in wearable healthcare systems. Inf. Fusion.

[22] Nickel C., Busch C., Rangarajan S., Mobius M. Using hidden markov models for accelerometer-based biometric gait recognition. Proceedings of the 2011 IEEE 7th International Colloquium on Signal Processing and Its Applications (CSPA).

[23] Zhang Y., Pan G., Jia K., Lu M., Wang Y., Wu Z. (2014). Accelerometer-based gait recognition by sparse representation of signature points with clusters. IEEE Trans. Cybern..

[24] Gafurov D., Snekkenes E., Bours P. Gait authentication and identifification using wearable accelerometer sensor. Proceedings of the 2007 IEEE Workshop on Automatic Identifification Advanced Technologies.

[25] Xu C., He J., Zhang X., Wang C., Duan S. (2018). Template-matching-based detection of freezing of gait using wearable sensors. Procedia Comput. Sci..

[26] Bobic V.N., Djuric-Jovieic M.D., Radovanovic S.M., Dragaevic N.T., Kostic V.S., Popovic M.B. Challenges of stride segmentation and their implementation for impaired gait. Proceedings of the 40th Annual International Conference of the IEEE Engineering in Medicine and Biology Society (EMBC).

[27] Tran L., Hoang T., Nguyen T., Kim H., Choi D. (2021). Multi-Model Long Short-Term Memory Network for Gait Recognition Using Window-Based Data Segment. IEEE Access.

[28] Juefei-Xu F., Bhagavatula C., Jaech A., Prasad U., Savvides M. Gait-id on the move: Pace independent human identification using cell phone accelerometer dynamics. Proceedings of the 2012 IEEE Fifth International Conference on Biometrics: Theory, Applications and Systems (BTAS).

[29] Rastegari E., Azizian S., Ali H. Machine learning and similarity network approaches to support automatic classification of parkinson’s diseases using accelerometer-based gait analysis. Proceedings of the 52nd Hawaii International Conference on System Sciences (HICSS).

[30] Ortiz N., Hernandez R.D., Jimenez R., Mauledeoux M., Aviles O. (2018). Survey of biometric pattern recognition via machine learning techniques. Contemp. Eng. Sci..

[31] Damaševičius R., Vasiljevas M., Šalkevičius J., Woźniak M. (2016). Human activity recognition in AAL environments using random projections. Comput. Math. Methods Med..

[32] Watanabe Y. Influence of holding smart phone for acceleration-based gait authentication. Proceedings of the 2014 Fifth International Conference on Emerging Security Technologies (EST).

[33] Chan H., Zheng H., Wang H., Sterritt R., Newell D. Smart mobile phone based gait assessment of patients with low back pain. Proceedings of the 2013 Ninth International Conference on Natural Computation (ICNC).

[34] Li G., Huang L., Xu H. iwalk: Let your smartphone remember you. Proceedings of the 2017 4th International Conference on Information Science and Control Engineering (ICISCE).

[35] Nickel C., Wirtl T., Busch C. Authentication of smartphone users based on the way they walk using k-nn algorithm. Proceedings of the 2012 Eighth International Conference on Intelligent Information Hiding and Multimedia Signal Processing (IIH-MSP).

[36] Choi S., Youn I.H., LeMay R., Burus S., Youn J.-H. Biometric gait recognition based on wireless acceleration sensor using k-nearest neighbor classification. Proceedings of the 2014 International Conference on Computing, Networking and Communications (ICNC).

[37] Pratama F.I., Budianita A. Optimization of K-Nn Classification in Human Gait Recognition. Proceedings of the 2020 Fifth International Conference on Informatics and Computing (ICIC).

[38] Zeng M., Nguyen L.T., Yu B., Mengshoel O.J., Zhu J., Wu P., Zhang J. Convolutional neural networks for human activity recognition using mobile sensor. Proceedings of the 6th International Conference on Mobile Computing, Applications and Services (MobiCASE).

[39] Zhang L., Wu X., Luo D. Recognizing human activities from raw accelerometer data using deep neural networks. Proceedings of the 2015 IEEE 14th International Conference on Machine Learning and Applications (ICMLA).

[40] Nguyen K.-T., Vo-Tran T.-L., Dinh D.-T., Tran M.-T. Gait Recognition with Multi-region Size Convolutional Neural Network for Authentication with Wearable Sensors. Proceedings of the International Conference on Future Data and Security Engineering (FDSE).

[41] Hannink J., Kautz T., Pasluosta C.F., Gasmann K.-G., Klucken J., Eskofier B.M. (2016). Sensor-based gait parameter extraction with deep convolutional neural network. IEEE J. Biomed. Health Inform..

[42] Zou Q., Wang Y., Wang Q., Zhao Y., Li Q. (2020). Deep Learning-Based Gait Recognition Using Smartphones in the Wild. IEEE Trans. Inf. Forensics Secur..

[43] Aghdam H.H., Heravi E.J. (2017). Guide to Convolutional Neural Networks.

[44] Sonnenburg S., Rätsch G., Schäfer C., Schölkopf B. (2006). Large scale multiple kernel learning. J. Mach. Learn. Res..

[45] Abdi H., Williams L.J. (2010). Principal component analysis. Wiley Interdiscip. Rev. Comput. Stat..

[46] Ngo T.T., Makihara Y., Nagahara H., Mukaigawa Y., Yagi Y. (2014). The Largest Inertial Sensor-Based Gait Database and Performance Evaluation of Gait-Based Personal Authentication. Pattern Recognit..

[47] Huang G., Liu Z., van der Matten L., Weinberger K.Q. Densely connected convolutional networks. Proceedings of the IEEE Conference on Computer Vision and Pattern Recognition (CVPR).

[48] Nair V., Hinton G.E. Rectified linear units improve restricted boltzmann machines. Proceedings of the 27th International Conference on Machine Learning (ICML).

[49] Fu J., Liu J., Tian H., Li Y., Bao Y., Fang Z., Lu H. Dual Attention Network for Scene Segmentation. Proceedings of the IEEE Conference on Computer Vision and Pattern Recognition (CVPR).

[50] Albahli S., Nida N., Irtaza A., Yousaf M.H., Mahmood M.T. (2020). Melanoma Lesion Detection and Segmentation Using YOLOv4-DarkNet and Active Contour. IEEE Access.

[51] Hu J., Shen L., Albanie S., Sun G., Wu E. Squeeze-and-Excitation Networks. Proceedings of the IEEE Conference on Computer Vision and Pattern Recognition (CVPR).

[52] Woo S., Park J., Lee J.-Y., Kweon I.S. Cbam: Convolutional block attention module. Proceedings of the European conference on computer vision (ECCV).

[53] Prechelt L. (1998). Automatic early stopping using cross validation: Quantifying the criteria. Neural Netw..

[54] Laurens V.D.M., Hinton G. (2008). Visualizing Data Using t-SNE. J. Mach. Learn. Res..

